# Supplementary material for: Relationships between putative G-quadruplex-forming sequences, RecQ helicases, and transcription
Source: BMC Med Genet. 2015 Oct 8;16:91. doi: 10.1186/s12881-015-0236-4 (PMC4599794; doi:10.1186/s12881-015-0236-4)
Supplement: Additional file 23: — Supplemental Figures S1 to S3 and Tables S1 to S7 with corresponding legends. (PDF 677 kb) [file 12881_2015_236_MOESM23_ESM.pdf]

Supporting Online Material for

Putative G-quadruplex forming sequence signatures in genes differentially transcribed upon loss of BLM or WRN helicases

John Smestad[1] and L. James Maher III[2]

[1]Mayo Clinic Medical Scientist Training Program

[2]Department of Biochemistry and Molecular Biology

Mayo Clinic College of Medicine

200 First St. SW, Rochester, MN 55905, USA

*To whom correspondence should be addressed. E-mail:.maher@mayo.edu

This PDF file includes:

Figures S1 to S3

Tables S1 to S7

Figure S1. Overlap Analysis of Genes Differentially-Expressed in BS and WS 3
Table S1. PQS Prevalence in Promoters of BS and WS Differentially-Expressed Genes 4
Figure S2. PQS Abundance Histograms of BS and WS Differentially-Expressed Genes 5
Table S2. Positions of PQS Excess/Scarcity in BS and WS Differentially-Expressed Genes 6
Table S3. Statistics for Analysis of PQS Abundance in BS and WS Differentially-Expressed Genes. 7
Figure S3. Epigenomic Track Correlations with Gene Expression 8
Table S4. Statistics for Analysis of PQS Genome-Wide Transcriptional Effects 9
Table S5. Statistics for Correlation of Epigenomic Tracks with Gene Expression. 10
Table S6. Bayesian Linear Regression Modeling Parameters 11
Table S7. Analysis of PQS Motifs in BS and WS Differentially-Expressed Genes 12

Figure S1. Overlap analysis of genes differentially-expressed in BS and WS.

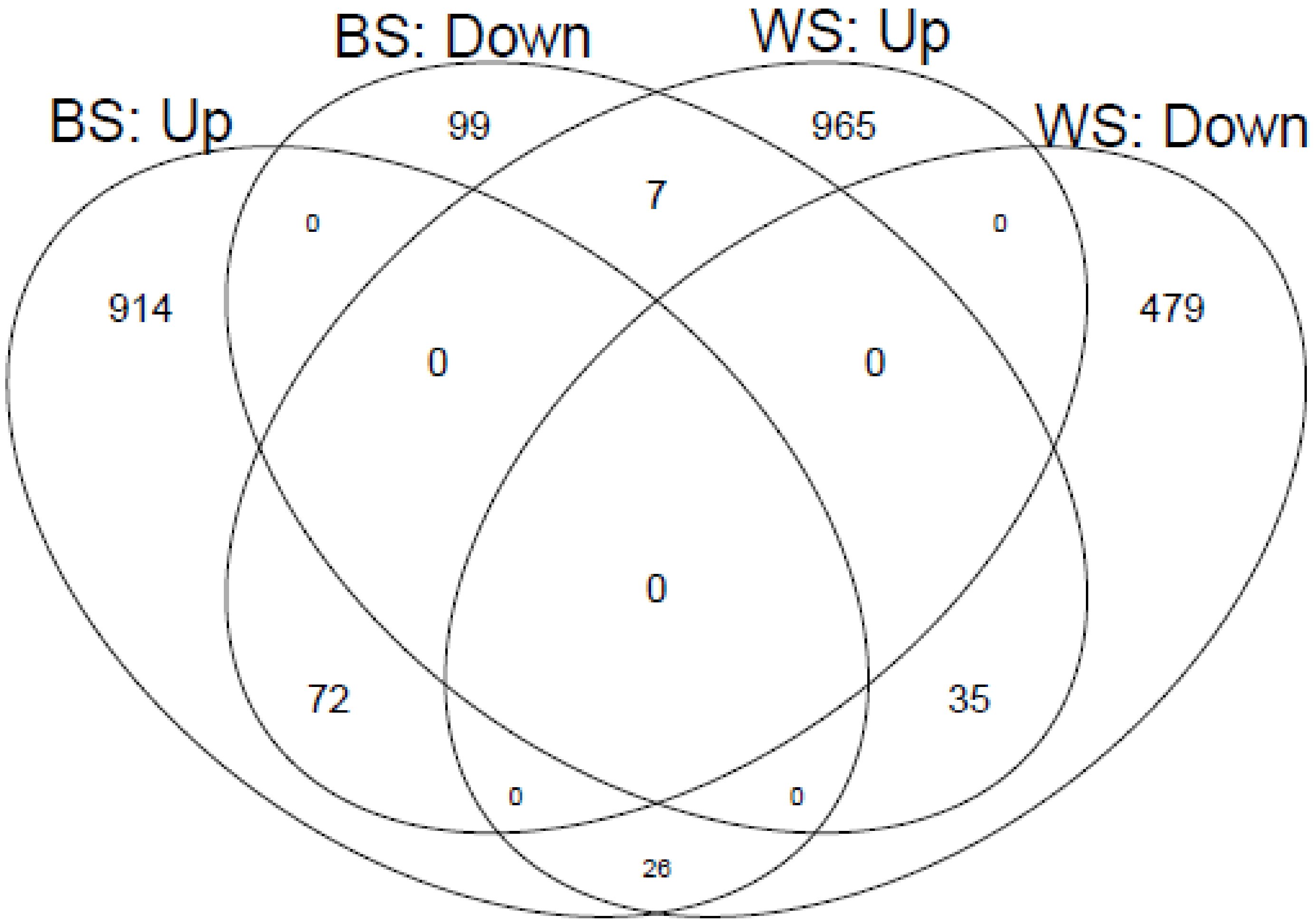

Table S1. PQS prevalence in promoters of BS and WS differentially-expressed genes.

		Bloom Syndrome		Werner Syndrome	
	**Whole Genome**	**Up-Regulated**	**Down-Regulated**	**Up-Regulated**	**Down-Regulated**
**Total Genes**	50541	1012	141	1046	540
**Genes Overlapping GENOCDE v7 Annotations**	50541	967	134	919	498
**% Genes Overlapping GENCODE v7 Annotations**	100	96	95	88	92
**GENCODE Genes with ≥ 1 PQS**	27627	817	121	678	417
**GENCODE Genes with No PQS**	22914	150	13	241	81
**% GENCODE Genes with PQS**	55	84	90	74	84

Figure S2. PQS abundance histograms of BS and WS differentially-expressed genes. Plots show positions of PQS near gene promoters genome-wide and in the subset of genes differentially expressed in BS and WS, assigned to sense (S) and antisense (AS) strand components. Histogram counts represent a 100-bp bin width spanning 2 kbp upstream and downstream of the TSS.

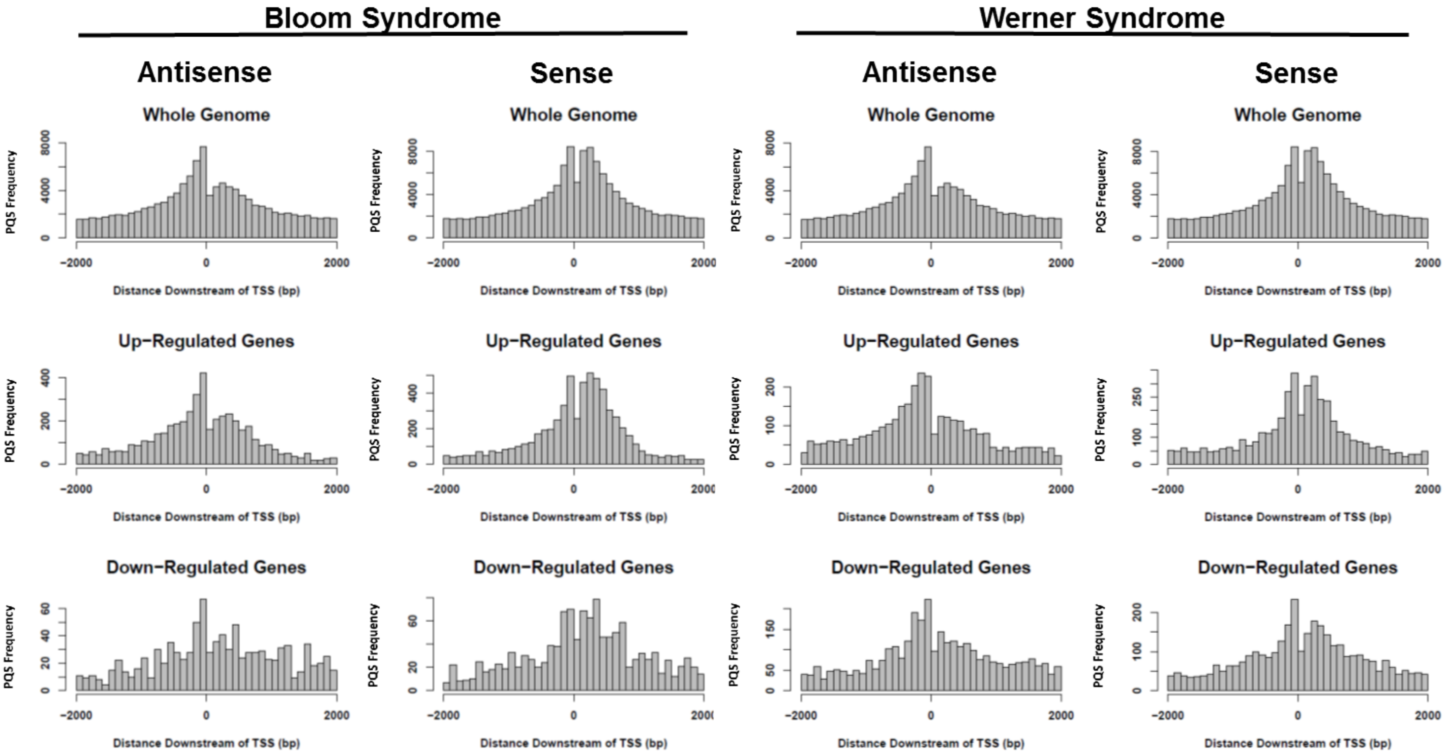

Table S2. Positions of statistically-significant PQS abundance excess and scarcity near the promoters of genes differentially expressed in BS and WS. Analysis is by antisense (AS) and sense (S) strands. Numbered regions correspond to the midpoint location of 200-bp bins used to compare numbers of PQS per TSS between differentially-expressed genes and the remainder of the human genome. All regions satisfy the criterion that the p-value is lower than the threshold p-value determined by statistical bootstrapping simulation using random gene sets.

**BS Up-Regulated: Antisense**	
**Enrichment:**	
**Depletion:**	-1900:-1870, -1800:-1780, -1750:-1690, -1640:-1630, -1450, -1430:-1260, 890:910, 1010:1900
**BS Up-Regulated: Sense**	
**Enrichment:**	-60:-50, -30, 160:680
**Depletion:**	-1900:-1100, -1080, 990:1900
**BS Down-Regulated: Anisense**	
**Enrichment:**	370, 450, 1090:1280, 1490:1610, 1710:1840
**Depletion:**	
**BS Down-Regulated: Sense**	
**Enrichment:**	-1360, -950:-940, -880:-870, -230:-210, -190:-80, 130, 280:320, 340, 360:430, 530:550, 570:830, 1010, 1030:1090, 1130:1260, 1670, 1690:1800
**Depletion:**	
**WS Up-Regulated: Antisense**	
**Enrichment:**	
**Depletion:**	10:70, 140:170, 320, 920:1360
**WS Up-Regulated: Sense**	
**Enrichment:**	
**Depletion:**	1490:1790
**WS Down-Regulated: Antisense**	
**Enrichment:**	-710:-520, -370:270, 300:380, 400:1040, 1130:1190, 1250:1840, 1870:1890
**Depletion:**	
**WS Down-Regulated: Sense**	
**Enrichment:**	-840:-560, -270, -250:-230, -190:-40, 830:930, 990:1080, 1100:1110, 1130:1160, 1310:1450
**Depletion:**	

Table S3. Statistics for analysis of PQS abundance in BS and WS differentially-expressed genes.

			Bloom Syndrome			Werner Sydnrome	
	Genome-Wide		Up-Regulated	Down-Regulated		Up-Regulated	Down-Regulated
**Genes**	50541		1012	141		1046	540
**TSS in Gene Dataset**	139700		6814	794		4850	2764
**PQS within 2 kb of a TSS**	251386		10963	2227		7685	6680
**Average Number PQS per TSS**	1.80		1.61	2.80		1.58	2.42
**Analysis of Antisense Strand:**							
**False Discovery Bin Fraction**	-		0.01	0.01		0.01	0.01
**Threshold P-Value**	-		1.1E-05	1.5E-07		5.7E-06	6.1E-06
**Predicted Number of False Discoveries**	-		3.81	3.81		3.81	3.81
**Number of Discoveries in Dataset**	-		128	49		57	229
**False Discovery Rate**	-		0.03	0.08		0.07	0.02
**Analysis of Sense Strand:**							
**False Discovery Bin Fraction**	-		0.01	0.01		0.01	0.01
**Threshold P-Value**	-		3.9E-06	2.3E-07		1.1E-06	3.5E-06
**Predicted Number of False Discoveries**	-		3.81	3.81		3.81	3.81
**Number of Discoveries in Dataset**	-		230	100		31	91
**False Discovery Rate**	-		0.02	0.04		0.12	0.04

Figure S3. Epigenomic track correlations with gene expression. Log_2-transformed epigenomic track feature correlations with log_2-transformed gene expression. The "bestbin" location for a particular feature was determined as the location with the highest absolute value correlation with expression. Bins were 100 bp in width and covered end-to-end the 1 kbp upstream and downstream of the TSS, giving a total of 20 bins. For the generation of this figure, a pseudocount equal to 0.25% of the maximal bin value was added to epigenetic track signals in order to avoid the $log_2(0)$ issue.

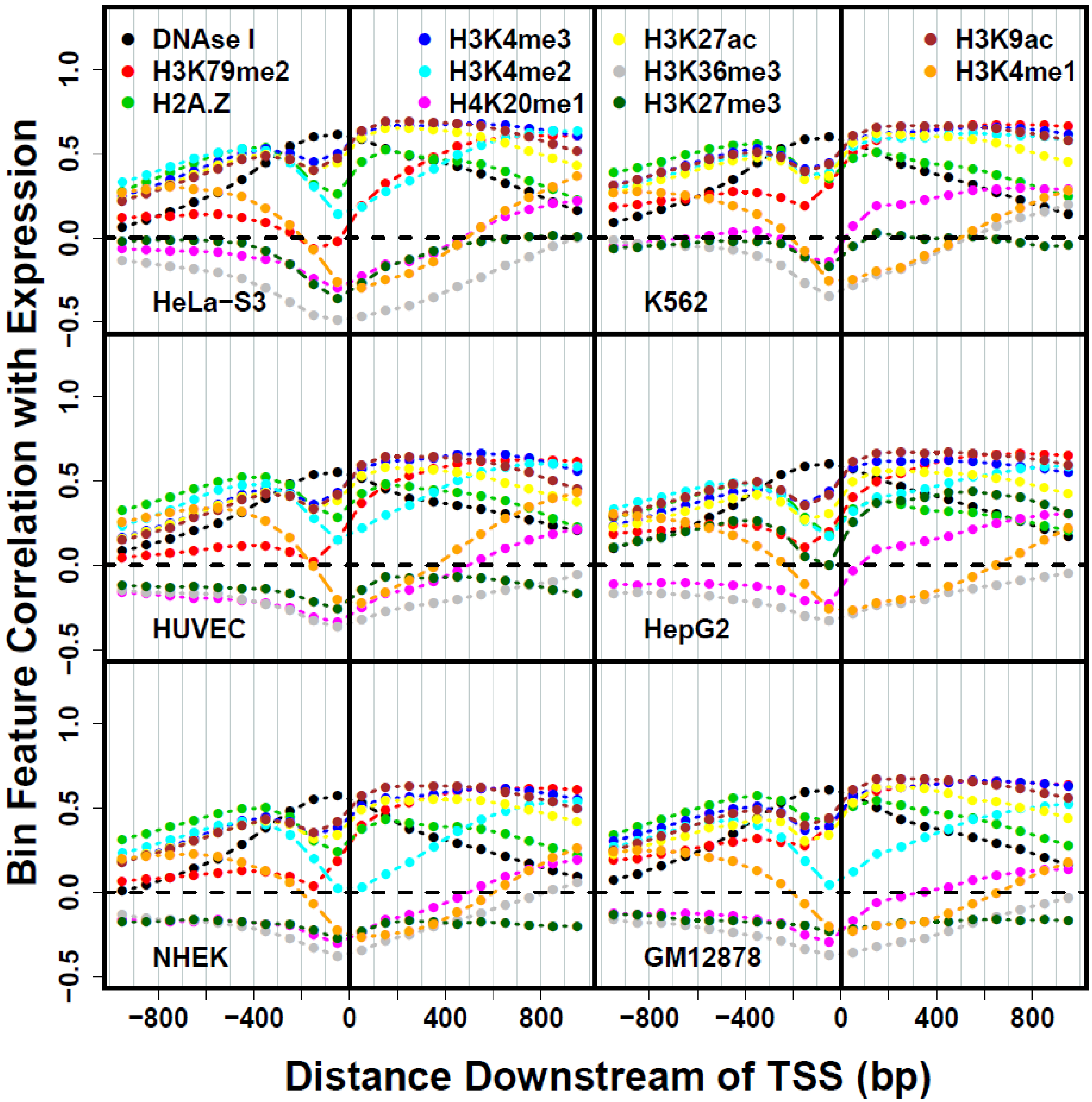

Table S4. Statistics for analysis of PQS genome-wide transcriptional effects.

	H1-hESC	HeLa-S3	K562	HUVEC	HepG2	NHEK	GM12878
**Total TSS in Dataset**	9614	6909	7484	5578	7659	4011	4447
**TSS with ≥ 1 PQS**	6919	4172	5076	3655	5416	2728	2974
**TSS with no PQS**	2695	2737	2408	1923	2243	1283	1473
**TSS where Nearest PQS is on TS**	3121	1849	2272	1616	2388	1181	1302
**TSS where Nearest PQS is on Non-TS**	3798	2323	2804	2039	3028	1547	1672
**Bayesian Modeling Correlation**	0.72	0.75	0.70	0.68	0.64	0.63	0.64
**Bayesian Modeilng RMSE**	2.9	3.0	3.0	3.1	2.9	3.2	3.2
**Analysis of Antisense Strand:**							
**False Discovery Bin Fraction**	0.01	0.01	0.01	0.01	0.01	0.01	0.01
**Threshold P-Value**	0.017	0.014	0.015	0.012	0.017	0.010	0.014
**Predicted Number of False Discoveries**	3.81	3.81	3.81	3.81	3.81	3.81	3.81
**Number of Discoveries in Dataset**	77	70	97	50	23	39	8
**False Discovery Rate**	0.049	0.054	0.039	0.076	0.166	0.098	0.476
**Analysis of Sense Strand:**							
**False Discovery Bin Fraction**	0.01	0.01	0.01	0.01	0.01	0.01	0.01
**Threshold P-Value**	0.012	0.012	0.011	0.011	0.014	0.015	0.015
**Predicted Number of False Discoveries**	3.81	3.81	3.81	3.81	3.81	3.81	3.81
**Number of Discoveries in Dataset**	57	55	53	13	33	46	7
**False Discovery Rate**	0.067	0.069	0.072	0.293	0.115	0.083	0.544

Table S5. Correlations of GC fraction and “bestbin” $\log_2$-transformed epigenetic tracks with $\log_2$-transformed gene expression. GC fraction is calculated for 5 kbp upstream of each TSS. Epigenetic track correlation values in this table represent the highest absolute value correlations determined by selecting both optimal bin and pseudocount for each track. Bins were 100 bp in width and covered end-to-end the 1 kbp upstream and downstream of the TSS, giving a total of 20 bins. Pseudocounts ranging from 0.25-4% of the maximal bin value were added to each bin prior to $\log_2$ transformation in order to avoid the $\log_2(0)$ issue.

	H1-hESC		HeLa-S3		K562		HUVEC		HepG2		NHEK		GM12878	
	**Cor.**	**P-Value**	**Cor.**	**P-Value**	**Cor.**	**P-Value**	**Cor.**	**P-Value**	**Cor.**	**P-Value**	**Cor.**	**P-Value**	**Cor.**	**P-Value**
**GC Fraction**	0.01	0.62	0.03	0.01	0.00	0.78	0.04	0.00	0.02	0.15	-0.01	0.65	-0.01	0.51
**DNAse 1**	0.46	0.00	0.65	0.00	0.57	0.00	0.50	0.00	0.50	0.00	0.50	0.00	0.53	0.00
**H3K79me2**	0.58	0.00	0.59	0.00	0.62	0.00	0.54	0.00	0.56	0.00	0.51	0.00	0.52	0.00
**H2A.Z**	0.43	0.00	0.58	0.00	0.51	0.00	0.47	0.00	0.36	0.00	0.40	0.00	0.48	0.00
**H3K4me3**	0.64	0.00	0.70	0.00	0.62	0.00	0.62	0.00	0.57	0.00	0.53	0.00	0.56	0.00
**H3K4me2**	0.52	0.00	0.64	0.00	0.59	0.00	0.53	0.00	0.48	0.00	0.45	0.00	0.42	0.00
**H4K20me1**	-0.26	0.00	-0.31	0.00	0.23	0.00	-0.31	0.00	0.24	0.00	-0.28	0.00	-0.30	0.00
**H3K27ac**	0.59	0.00	0.68	0.00	0.58	0.00	0.53	0.00	0.51	0.00	0.46	0.00	0.52	0.00
**H3K36me3**	-0.28	0.00	-0.50	0.00	-0.32	0.00	-0.33	0.00	-0.28	0.00	-0.30	0.00	-0.33	0.00
**H3K27me3**	-0.28	0.00	-0.36	0.00	-0.19	0.00	-0.22	0.00	0.43	0.00	-0.20	0.00	-0.21	0.00
**H3K9ac**	0.64	0.00	0.71	0.00	0.62	0.00	0.61	0.00	0.58	0.00	0.53	0.00	0.57	0.00
**H3K4me1**	0.23	0.00	0.39	0.00	0.18	0.00	0.33	0.00	0.14	0.00	-0.32	0.00	0.10	0.00

Table S6. Bayesian linear regression modeling parameters for the seven human cell lines used in epigenetic modeling of gene expression.

	H1-hESC	HeLa-S3	K562	HUVEC	HepG2	NHEK	GM12878
**GC Fraction**	-0.039	-0.102	-0.141	-0.108	-0.111	0.024	-0.172
**DNAse 1**	0.272	0.273	0.492	0.222	0.187	0.354	0.207
**H3K79me2**	0.244	-0.010	0.422	0.290	-0.032	0.318	-0.148
**H2A.Z**	0.044	0.143	0.063	0.081	0.287	0.096	0.227
**H3K4me3**	0.257	0.077	0.053	-0.353	-0.049	0.000	-0.065
**H3K4me2**	-0.051	0.045	-0.142	0.263	-0.064	-0.071	0.019
**H4K20me1**	-0.124	-0.104	0.002	-0.133	0.017	0.140	0.209
**H3K27ac**	0.120	-0.177	0.134	-0.170	0.301	-0.092	0.014
**H3K36me3**	0.026	0.015	-0.078	-0.040	0.127	-0.034	0.075
**H3K27me3**	-0.133	-0.031	-0.076	0.015	-0.012	-0.125	0.010
**H3K9ac**	0.030	0.210	0.013	0.167	-0.042	0.076	0.028
**H3K4me1**	-0.029	0.010	-0.099	-0.014	0.054	0.065	0.047

Table S7. Analysis of PQS motifs in genes differentially expressed in BS and WS. (A) FDR for the binned analysis in Figure 4, with FDR calculated as the ratio of statistically-significant bins in BS and WS compared to the average statistically-significant bin number in randomly-generated datasets containing the same number of genes as the test datasets. (B) Average PQS feature values for whole genome sense (S) and antisense (AS) strands, and for the subset of genes up-regulated or down-regulated in BS and WS. Signals are averaged over the entire 2 kbp upstream and downstream of the TSS. Percent difference from the average genomic value is shown.

A)

False Discovery Rates								
	Fraction Adenine	Fraction Cytosine	Fraction Thymidine	Number of G Stacks	Loop 1 Length	Loop 2 Length	Loop 3 Legth	Total Length
**BS Up-Regulated: Antisense**	0.002	0.003	0.001	0.002	Inf	Inf	0.001	0.002
**BS Down-Regulated: Antisense**	0.003	0.006	0.002	0.002	0.030	Inf	Inf	0.003
**WS Up-Regulated: Antisense**	0.733	Inf	0.111	Inf	0.553	0.385	0.086	0.235
**WS Down-Regulated: Antisense**	Inf	0.144	0.125	Inf	Inf	0.102	Inf	Inf
**BS Up-Regulated: Sense**	0.002	0.003	0.002	Inf	Inf	0.002	Inf	0.002
**BS Down-Regulated: Sense**	0.016	0.003	0.002	Inf	Inf	0.026	Inf	Inf
**WS Up-Regulated: Sense**	0.176	0.202	Inf	0.180	Inf	Inf	Inf	0.196
**WS Down-Regulated: Sense**	Inf	Inf	Inf	0.455	0.166	Inf	0.805	Inf

B)

	Fraction Adenine		Fraction Cytosine		Fraction Thymidine		Number of G Stacks		Loop 1 Length		Loop 2 Length		Loop 3 Legth		Total Length	
	Value	% Dif	Value	% Dif	Value	% Dif	Value	% Dif	Value	% Dif	Value	% Dif	Value	% Dif	Value	% Dif
**Whole Genome: Antisense**	0.113	-	0.127	-	0.082	-	4.729	-	3.465	-	3.423	-	3.448	-	29.44	-
**Whole Genome: Sense**	0.105	-	0.132	-	0.088	-	4.738	-	3.506	-	3.415	-	3.445	-	29.45	-
**BS Up-Regulated: Antisense**	0.117	3.6	0.122	-3.8	0.063	-22.7	4.604	-2.6	3.404	-1.8	3.604	5.3	3.236	-6.2	28.70	-2.5
**BS Down-Regulated: Antisense**	0.105	-7.4	0.121	-4.7	0.093	14.1	4.535	-4.1	3.201	-7.6	3.613	5.6	3.397	-1.5	27.94	-5.1
**WS Up-Regulated: Antisense**	0.116	2.4	0.129	1.5	0.075	-7.8	4.678	-1.1	3.621	4.5	3.606	5.3	3.843	11.5	28.93	-1.7
**WS Down-Regulated: Antisense**	0.115	1.3	0.124	-2.7	0.076	-6.7	4.701	-0.6	3.435	-0.8	3.158	-7.8	3.493	1.3	29.65	0.7
**BS Up-Regulated: Sense**	0.092	-12.1	0.142	8.0	0.076	-12.9	4.718	-0.4	3.493	-0.4	3.249	-4.9	3.438	-0.2	28.93	-1.8
**BS Down-Regulated: Sense**	0.107	1.5	0.127	-3.2	0.097	10.7	4.667	-1.5	3.818	8.9	3.089	-9.5	3.395	-1.4	29.44	0.0
**WS Up-Regulated: Sense**	0.100	-5.2	0.137	4.1	0.085	-2.6	4.658	-1.7	3.407	-2.8	3.509	2.8	3.538	2.7	28.67	-2.6
**WS Down-Regulated: Sense**	0.104	-1.0	0.132	0.4	0.086	-1.6	4.677	-1.3	3.277	-6.5	3.32333	-2.7	3.605	4.6	29.27	-0.6
